# Lipid profile and risk of ovarian tumours: a meta-analysis

**DOI:** 10.1186/s12885-020-6679-9

**Published:** 2020-03-12

**Authors:** Justina Ucheojor Onwuka, Akinkunmi Paul Okekunle, Olaniyi Matthew Olutola, Onoja Matthew Akpa, Rennan Feng

**Affiliations:** 1 Department of Epidemiology, College of Public Health, Harbin Medical University, 157 Baojian Street, Harbin, Heilongjiang Province 150081 People’s Republic of China; 2 Department of Nutrition and Food Hygiene, College of Public Health, Harbin Medical University, 157 Baojian Street, Harbin, Heilongjiang 150081 People’s Republic of China; 3 Department of Epidemiology and Medical Statistics, College of Medicine, University of Ibadan, Ibadan, 200284 Nigeria; 4 The Postgraduate College, University of Ibadan, Ibadan, 200284 Nigeria; 5 Institute of Cardiovascular Diseases, College of Medicine, University of Ibadan, Ibadan, 200284 Nigeria

**Keywords:** Lipid profile, Total cholesterol, Triglyceride, High-density lipoprotein, Low-density lipo-protein, Ovarian tumour

## Abstract

**Background:**

Existing data from several reports on the association between lipid profile and ovarian tumour (OT) suggests divergent conclusions. Our aim was to examine whether circulating lipid profile: total cholesterol (TC), triglyceride (TG), high-density lipoprotein (HDL) and low-density lipoprotein (LDL) differed between cases and non-cases of OT.

**Methods:**

Electronic repositories; PUBMED, EMBASE and Cochrane library were explored through December 2019 to retrieve published articles for inclusion in the meta-analysis after quality assessment. Heterogeneity was assessed using *I*^*2*^ statistics, the effect of individual studies on the overall effect size was tested using sensitivity analysis and funnel plot was used to evaluate publication bias.

**Results:**

Twelve studies, involving 1767 OT cases and 229,167 non-cases of OT were included in this meta-analysis and *I*^*2*^ statistics ranged between 97 and 99%. Mean circulating TC (− 16.60 [− 32.43, − 0.77]*m*g/*d*L; *P* = 0.04) and HDL (− 0.25[− 0.43, − 0.08]*m*mol/L; *P* = 0.005) were significantly lower among OT cases compared to non-OT cases.

**Conclusion:**

Decreased TC and HDL profiles were observed among subjects with OT in this collection of reports. The implications of TC and HDL in tumour manifestations and growth need to be validated in a large multi-ethnic longitudinal cohort adjusting for relevant confounders.

## Introduction

Ovarian cancer is the most deadly gynaecological malignancy among women, comprising diverse groups of neoplasm [1]. It accounts for 2.3% of all cancer-related death in the US [2], 4% of all new cancer cases among women, the fifth commonest cancer and the fourth cause of malignancy-related death in the UK [3]. Lipids are biologically-important hydrophobic molecules vital for energy storage, cell signalling, maintenance of cell membrane integrity [4] and are transported in the bloodstream with the aid of lipoprotein [5].

Several studies have reported the relationship between lipid profiles; total cholesterol (TC), triglycerides (TG), high-density lipoprotein cholesterol (HDL) and low-density lipoprotein cholesterol (LDL) and ovarian tumour (OT) with different conclusions. For example, Camuzcuoglu et al. [6] and Bukhari et al. [7] in separate reports observed TC was significantly lower among OT patients compared to healthy controls. Contrariwise, Melvin et al. [8] observed no difference in circulating TC profiles between cases and non-cases of OT. Furthermore, Gadomska et al. [9] and Camuzcuoglu et al. [6] found HDL profile was lower among OT patients compared to healthy controls. Whereas, Delimaris et al. [10] and Melvin et al. [8] found no association between HDL and OT risk.

Drawing vivid inferences from prior population-based studies on lipid profile and OT risk appears difficult because of disparities in participants’ selections, study designs, etc. In addition, scientific evidence on this subject is of great significance to clarify whether alterations in circulating lipid profiles are sufficient to promote OT risk or these alterations are only a reflection of previously compromised health status.

To this effect, a comprehensive analysis, comprising previous studies across diverse population would be necessary. Therefore, this study investigated the true difference in circulating lipid profiles (TC, TG, HDL and LDL) among subjects with and without OT using a meta-analytical approach.

## Materials and methods

**Fig. 1:**
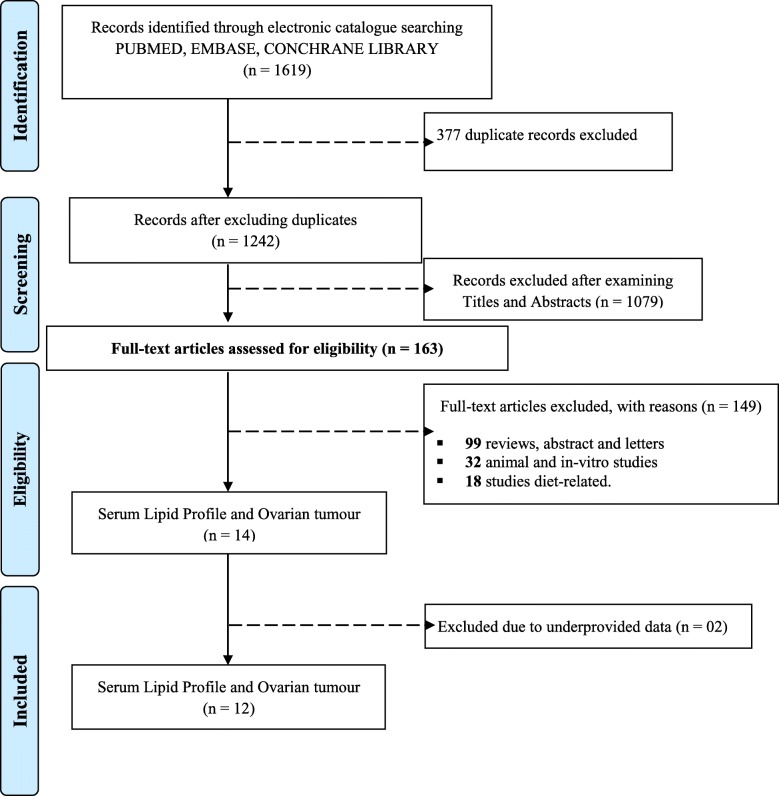
PRISMA flowchart for inclusion and exclusion of studies in the meta – analyses

This meta-analysis was prospectively registered on PROSPERO (https://www.crd.york.ac.uk/PROSPERO/display_record.php?ID=CRD42018099728) and conducted using the MOOSE guidelines [11, 12]. Electronic scientific repositories; PubMed, EMBASE and Cochrane Library were extensively searched (without language and period of publication restrictions) through December 2019 to identify published studies using the following keywords: “lipid profile” OR “total cholesterol” OR “triglycerides” OR “high-density lipoprotein” OR “low-density lipoprotein” AND “ovarian cancer” OR “ovarian carcinoma” OR “epithelial ovarian cancer” OR “ epithelial ovarian carcinoma” OR “ovarian benign tumour” OR “ovarian malignant tumour” OR “ovarian tumour”. Also, references of retrieved articles were searched manually for more studies and PRISMA flowchart explaining the search methodology is shown in Fig. 1. In addition, evaluation of titles and abstracts of retrieved articles were independently done by two reviewers and difference(s) were addressed in consultation with a third reviewer.

### Study selection

A study is included in the meta-analysis if it; (a) is a case-control studies in human population that investigated the association between lipid profiles and ovarian tumour, (b) compared cases (women with ovarian tumour) with non-cases (women without ovarian tumour) and (c) reported lipid profile (TC and/or HDL and/or LDL and/or TG) in bloodstream in at least two groups (cases and non-cases) for comparison in a singular study. Similar reports among pregnant and lactating women, animals and cell lines were excluded. Also, abstracts, reviews, letter to the editor and conference papers were excluded.

### Quality assessment of studies

The methodological quality and risk of bias of studies included in the meta-analysis were assessed using the Cochrane Collaboration guidelines and the Newcastle-Ottawa scale [13]. Briefly, two reviewers independently appraised the quality of studies and dissimilarities were conciliated by a third reviewer.

### Data extraction

Name of authors, year of publication, country, study population, sample size, lipid profile(s) determined, methods of analysis, criteria for case definition, mean values [with standard deviation (SD), standard error of mean (SEM), confidence interval (CI)] of serum lipid profile (TC, HDL, LDL and TG) were extracted independently by two reviewers and differences in data extractions were resolved in recourse to a third reviewer. To ensure uniformity of estimates, mean values of TC were transformed to (mg/*dL*), but TG, HDL and LDL were transformed to (mmol/*L*). Also, all values reported as SEM and CI were transformed into SD [14].

### Statistical analysis

Heterogeneity of pooled effect estimate and the magnitude of variation across studies was assessed using *I*^*2*^ test statistics. A random-effects model was used to obtain mean estimates under considerable heterogeneity (i.e *I*^*2*^-test > 50% or *P* < 0.05), but a fixed-effect model was applied to obtain mean estimates when *I*^*2*^-test < 50% or *P* > 0.05. The random effect model postulates mean estimates of lipid profile(s) differed across studies, but follow a distribution and pooled mean is estimated as the average mean difference with an assumption that differences in mean estimates are symmetrically distributed. However, the fixed effect estimates assumed that observed differences are primarily an after-effect of chance [12, 15].

Statistical analysis was conducted using Review Manager 5.3 and two-tailed *P < 0.05* was considered statistically significant. Sensitivity analysis of pooled mean estimates was assessed using a leave-one-out method and publication bias was assessed using a funnel plot.

## Results

### Literature search

Of the 1619 records obtained from the primary literature search, 377 duplicates and 1079 records were excluded after examining titles and abstracts. Also, 151 records were excluded after full-text evaluation and 12 studies [6–10, 16–22] comprising 1767 OT cases and 229,167 non-cases of OT met the inclusion criteria (Fig. 1). Characteristics of the included studies are shown in Table 1.

**Table 1:** Characteristics of all eligible studies for lipid profile and risk of ovarian tumours

Authors	Year	Country	Cases	Control	Lipid profile^a^	Ascertainment of ovarian tumour cases	Classification^f^	Accountability of bias
Bukhari et al. [7]	2016	Pakistan	30	30^e^	TC, TG, HDL, LDL^b^	Hospital/Medical record confirmed using color flow Doppler tests, biopsies and MRI	NR	NR
Camuzcuoglu et al. [6]	2009	Turkey	24	29^e^	TC, TG, HDL, LDL^b^	Hospital/Medical record	FIGO	Excluded^g, h^
Chen et al. [16]	2017	China	573	1146^d^	TG, HDL^b^	Hospital confirmed	FIGO	Excluded^h,i^
Das et al. [17]	1987	China	28	66^e^	TC^b^	Histopathological examinations	NR	NR
Delimaris et al. [10]	2007	Greece	15	30^d^	TC, HDL, LDL^b^	Hospital/Medical records,	TNM	Excluded^g^
Gadomska et al. [18]	1997	NR	25	25^e^	TC, TG, HDL^b^	Histopathological examinations	FIGO	NR
Gadomska et al. [9]	2005	Poland	91	44 ^e^	TC, TG, HDL^b^	Histopathological examinations, Transvaginal ultrasonography,	FIGO	NR
Knapp et al. [19]	2017	Poland	74	81^e^	TC, TG	Transvaginal sonography evaluation, Histopathological examinations, CT scan	FIGO	Excluded^h,i^
Kuesel et al. [20, 23]	1992	Canada	62	51^e^	TC, TG	NR	FIGO	NR
Melvin et al [8]	2012	Sweden	786	227,603^d^	TC, TG, HDL, LDL^b^	Verifiable database	NR	NR
Qadir et al. [21]	2008	Pakistan	40	50^d^	TC, TG, HDL, LDL^b^	NR	NR	Excluded^h^
Yam et al. [22]	1994	Israel	19	12^c^	TC, TG, HDL, LDL^b^	Biopsies of ovary/endometrium	NR	NR

NR-not reported; ^a^-lipid profile reported in the study; ^b^-lipid profile assessed in fasting state; ^c^-hospital-based controls; ^d^-population-based controls ^e^-unspecified type of controls; MRI-magnetic resonance imaging; ^f^-method adopted for tumour classification; FIGO-International Federation of Gynecologists and Obstetricians; TNM-The TNM Classification of Malignant Tumours

^g^Patients with previously-performed chemo-therapy, radiotherapy and surgery

^h^Patients with concurrent or previous malignant disease or any other disease

^i^Patients with suspected abnormalities such as neoplastic effects etc

#### Pooled mean difference of circulating TC, TG, LDL, HDL between OT and non-OT subjects

**Fig. 2:**
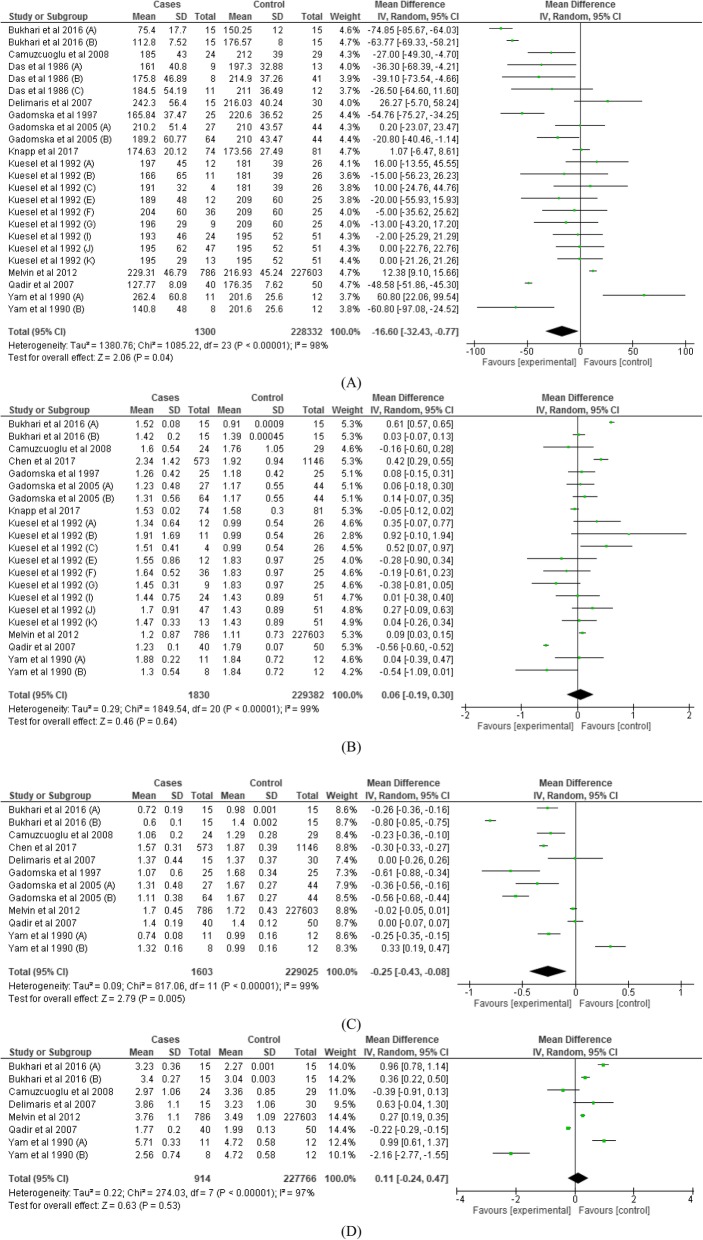
Forest plot of lipid profile; total cholesterol (**a**), triglyceride (**b**), HDL (**c**) and LDL (**d**) between cases and non-cases of ovarian tumour

Mean TC; − 16.60 [− 32.43, − 0.77]*m*g/*d*L, *P* = 0.04 was significantly lower among OT cases compared to non-OT subjects (Table 2 and Fig. 2). Similarly, mean HDL; − 0.25 [− 0.43, − 0.08]*m*mol/*L*, *P* = 0.005 was significantly lower among OT cases compared to non-OT subjects. However, these differences were insignificant after stratifying by age groups. Also, mean TG and LDL differed insignificantly between OT and non-OT subjects.

**Table 2:**
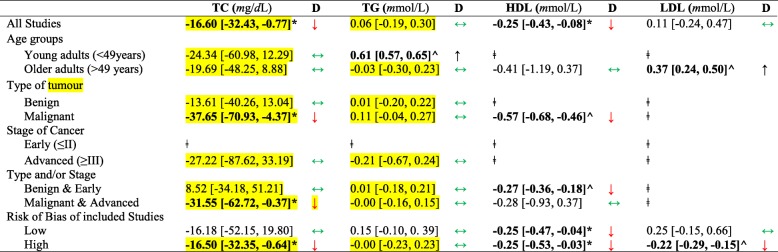
Mean difference and 95% CI of Lipid Profile between cases and non-cases of ovarian tumours

D-direction of mean difference relative to non-ovarian tumour cases; TC-Total cholesterol; TG-Triglycerides; HDL-High density lipoprotein; LDL-Low density lipoprotein

*p < 0.05

**^***p* < 0.00001

studies were insufficient to carry out the meta-analysis

mean difference significantly higher among cases than non-cases of ovarian tumour

mean difference significantly lower among cases than non-cases of ovarian tumour

mean difference insignificantly different between cases than non-cases of ovarian tumour

Stratifying our meta-analysis by age (Table 2), TG profile was significantly elevated; 0.61 [0.57, 0.65]*m*mol/*L P* < 0.0001 among OT subjects < 49 years only. Contrariwise, LDL profile was significantly elevated; 0.37 [0.24, 0.50]*m*mol/*L P* < 0.0001 among OT subjects > 49 years only. TC was significantly lower (− 31.55 [− 62.72, − 0.37] mg/*dL P < 0.05*) among OT subjects with malignant and/or advanced tumours. TC and LDL profiles were insignificantly different, but HDL profile was significantly lower between OT and non-OT subjects in studies with low risk of bias.

### Quality assessment and risk of bias

Fifty percent of studies included in the meta-analysis suggested a low risk of bias (Table S1). The bias observed in most studies was mostly attributed to incongruities in the case definition of OT (Figure S2).

### Publication bias

There was no significant evidence of publication bias from the funnel plots (Figure S1) in the meta-analysis.

### Sensitivity analysis

Overall pooled mean estimates differed insignificantly upon the exclusion of a single study at a time (Table S2), but few studies [7, 17, 18, 21, 22] exerted negligible influence on the overall pooled mean estimates of the meta-analysis.

## Discussion

To the best of our knowledge, this is the first meta-analytical study reporting true mean differences of circulating lipids and OT risk. Total cholesterol and HDL profiles were significantly lower among OT subjects, but TG and LDL profiles were insignificant in OT risk. We opined that these results highlight the significance of lipids in OT outcomes. The quality of reports included in our meta-analysis may largely influence these associations, but the findings of the stratified analysis represent a significant strength and modest evidence for significant alterations of lipid profile in OT risk is likely.

Whether altered lipid profiles are a causal or consequential factor of OT risk is debatable. However, our findings aligned with the plausibility of the latter. Circulating lipid profiles are largely subject to alterations in the occurrence of tumour events [24]. Cholesterol can be acquired from diet or endogenous biosynthesis and some studies [23, 25] have established the contributions of higher dietary cholesterol to OT risk. The occurrence of significantly lower circulating TC in OT subject may be preclinical and perhaps attributable to chronic exposure to higher cholesterol intakes. On the other hand, our findings appear consistent with other reports where TC was lower across several cancer sites [26–28]. Furthermore, the strong affinity of cancer cells for sterols and lipids makes lipid metabolism a critical factor in cancer signalling [29, 30]. For example, excessive production of lipogenic enzymes has been observed in several cancers [31] and is linked with cancer severity and reoccurrence [32, 33]. Also, increased signalling activity of a combination of steroid hormone receptors and growth factors via several complex metabolic circuits [34–36] modulate and activate SREBP-1 – the principal regulatory factor of lipogenesis in cancer cells.

HDL and LDL are prominent cholesterol-transporting agents vital in evaluating lipid profile in cancer signalling [29, 30]. In our study, we observed HDL (and not LDL) was inversely related to OT risk. The conventional purpose of HDL involves the assemblage of cholesterol from peripheral tissues for transportation to the liver for the purpose of excretion [37]. In tandem with our findings, Gadomska et al. [18] in a multidimensional analysis established lower concentrations of HDL sub-fractions of total cholesterol and esterified cholesterol significantly discriminated women with ovarian neoplasm. It is plausible that HDL (more than LDL) perhaps is the focal driver of the TC-OT risk link given the absence of an association between LDL and OT risk. From a clinical point of view, the pathophysiology of the inverse HDL-OT link is yet to be well understood. However, the high demand for cholesterol in cancers can as well impose the upregulation of scavenger receptor class B type 1 to mobilize HDL for increased cholesterol influx to promote proliferation and hormone synthesis for tumour cell growth and survival thereby leading to decrease in circulating HDL [38]. On this premise, it is not strange that the applicability and viability of lipoprotein-based nanoparticles drug delivery mechanism for cancer treatment have been reported in the literature [39–41]. For example, the biocompatibility, reliability and viability of engineered HDL nanoparticles conjugated with folic acid as carriers drug delivery targets to metastatic ovarian cancer sites in mouse models has been documented [39].

In addition, there is evidence of a modest inverse association between TC or HDL and breast cancer risk [28]. The anti-inflammatory properties of HDL in inhibiting cell proliferation and apoptosis [42] in addition to plummeting LDL oxidative potency in order to prevent increased intracellular oxidative stress is a critical step in cancer pathogenesis [43]. Decreased HDL levels are associated with increased levels of pro-inflammatory cytokines, including tumour necrosis factor-alpha and interleukin-6 [44].

Also, LDL differed insignificantly between OT and non-OT subjects in this current meta-analysis. This finding has been well reported in studies [28, 45] from other cancer sites. Tumour cells express increased LDL receptor levels which lead to low LDL levels [46]. LDL receptors are regulated by the SREBP transcriptional assembly [47] and can promote the intracellular influx of cholesterol to induce carcinogenesis. Conversely, excess cholesterol and its oxidized metabolites can activate liver X receptors and retinoid X receptors heterodimeric transcriptional factors to suppress LDL and induce ABC-family transporter expression to promote cholesterol efflux [48].

Our study has both strengths and limitations. Our report is the first meta-analysis highlighting the significance of lipid profile and risk of ovarian neoplasm. The higher statistical power arising from a large number of participants in our report potentially offer credibility to our findings. In addition, our funnel plots could not rule out the potential for publication bias in this meta-analysis. However, most studies included in our meta-analysis were cross-sectional (owing to limited cohort reports) and limited studies age-matched cases with controls in the eligible studies. The number of studies on this subject is comparatively rare, making the clarification of our findings quite challenging. Our findings must be interpreted with caution given a temporal sequence of causal association cannot be inferred and perhaps prone to reverse causality. In spite of the biological plausibility of the association between lipid profile and OT risk, there are many confounders involved in OT carcinogenesis. Overweight/obesity and its associated co-morbidities significantly promote OT risk among women [49]. Similarly, excessive weight gain is associated with features of metabolic syndrome and low circulating HDL levels [50]. Information regarding these confounders and comorbidities such as; diabetes, endometriosis, OT subtypes, weight status, smoking status, use of hormone replacement therapy or statin and/or fibrate treatment, etc. were relatively omitted in most reports included in our meta-analysis. Hence, prospective cohort studies adjusting for these confounders are recommended to validate the findings of this meta-analysis. Also, the bias of recall, selection and confounding is likely, but the quality assessment of studies and indifference in the overall our findings after a sensitivity analysis justifies the legitimacy of our results.

## Conclusion

Our meta-analysis presents evidence of a modest significant association between circulating HDL and risk of OT. It is vital to elucidate the implications of HDL in tumour manifestations and growth. There is a need to validate these findings using large multi-ethnic longitudinal cohorts effectively adjusting for age, menopausal status, preclinical prejudice and other key confounding factors.

## Supplementary information

**Additional file 1. Supplementary Table S1:** Critical assessment of included studies using the Newcastle-Ottawa Scale (NOS). **Supplementary Figure S1:** Funnel plot of lipid profile; total cholesterol (A), triglyceride (B), HDL (c) and LDL (D) between cases and non-cases of ovarian tumours; summarizing the publication bias in the meta-analysis. **Supplementary Figure S2:** Graphical illustration of the results of the critical assessment of studies; Is the Case Definition Adequate? (S1), representativeness of the Cases (S2), selection of Controls (S3), definition of Controls (S4), comparability of cases and controls on the basis of the design or analysis (C1), ascertainment of exposure (E1), same method of ascertainment of exposure for cases and controls (E2), overall risk of bias of all studies included. **Supplementary Table S2:** Sensitivity Analysis (using one study leave out method) of pooled mean differences of Lipid profiles between cases and non-cases of ovarian tumour.

**Additional file 2.** A Meta-analysis Of Observational Studies in Epidemiology (MOOSE) Checklist.

## Data Availability

The dataset(s) supporting the conclusions of this article is(are) included within the article (and its additional file(s)).

## Abbreviations

CI: Confidence interval
HDL: High-density lipoprotein
LDL: Low-density lipoprotein
OT: Ovarian tumour
SD: Standard deviation
SEM: Standard error of the mean
TC: Total cholesterol
TG: Triglyceride
