# Supplementary material for: Lipid profile and risk of ovarian tumours: a meta-analysis
Source: BMC Cancer. 2020 Mar 12;20:200. doi: 10.1186/s12885-020-6679-9 (PMC7068873; doi:10.1186/s12885-020-6679-9)
Supplement: Supplementary file 1 — Additional file 1. Supplementary Table S1: Critical assessment of included studies using the Newcastle-Ottawa Scale (NOS). Supplementary Figure S1: Funnel plot of lipid profile; total cholesterol (A), triglyceride (B), HDL (c) and LDL (D) between cases and non-cases of ovarian tumours; summarizing the publication bias in the meta-analysis. Supplementary Figure S2: Graphical illustration of the results of the critical assessment of studies; Is the Case Definition Adequate? (S1), representativeness of the Cases (S2), selection of Controls (S3), definition of Controls (S4), comparability of cases and controls on the basis of the design or analysis (C1), ascertainment of exposure (E1), same method of ascertainment of exposure for cases and controls (E2), overall risk of bias of all studies included. Supplementary Table S2: Sensitivity Analysis (using one study leave out method) of pooled mean differences of Lipid profiles between cases and non-cases of ovarian tumour. [file 12885_2020_6679_MOESM1_ESM.docx]

| 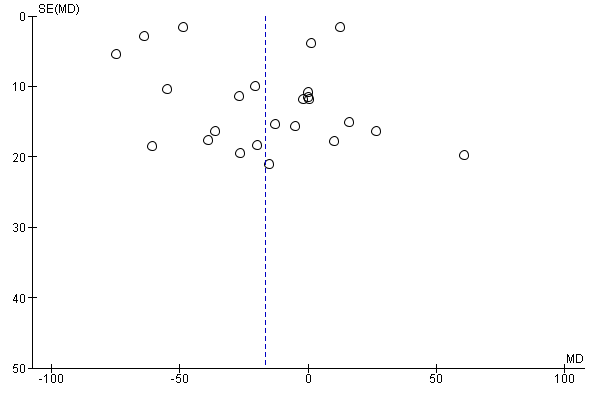 | 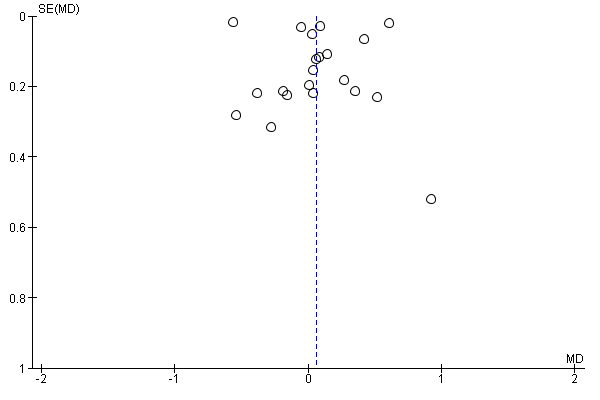 |
| --- | --- |
| A | B |
| 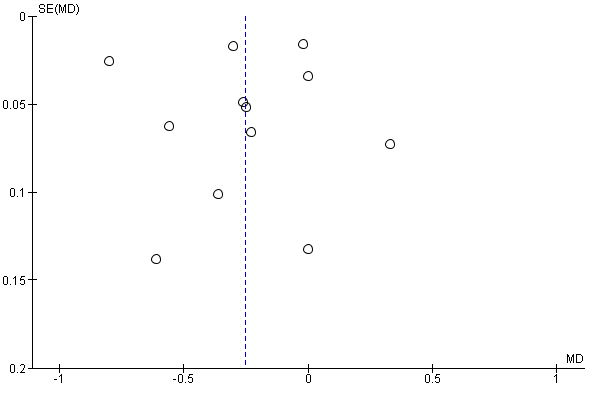 | 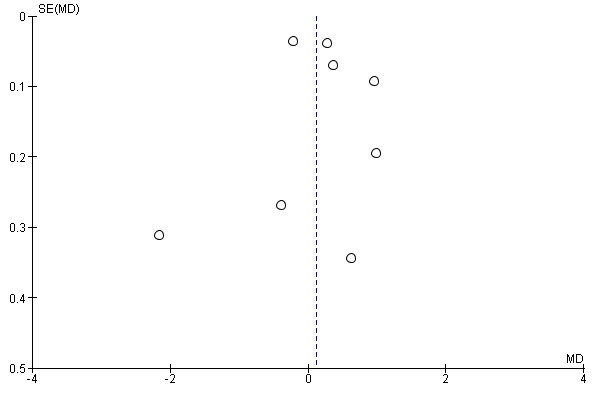 |
| C | D |
| Supplementary Figure S1: Funnel plot of lipid profile; total cholesterol (A), triglyceride (B), HDL (c) and LDL (D) between cases and non-cases of ovarian tumours. | |

| Supplementary Table S1: Critical assessment of included studies using Newcastle-Ottawa Scale (NOS) | | | | | | | | | | | | | | | |
| --- | --- | --- | --- | --- | --- | --- | --- | --- | --- | --- | --- | --- | --- | --- | --- |
|  |  | Selection | | | |  | Comparability |  | Exposure | | |  | Total Score |  | ROB |
| Study | Year | S1 | S2 | S3 | S4 |  | C1 |  | E1 | E2 | E3 |  |  |  |  |
| Bukhari et al | 2016 | * | * |  | * |  | * |  | * | * |  |  | 6 |  | Low |
| Camuzcuoglu et al | 2008 |  | * |  | * |  | * |  | * | * |  |  | 5 |  | High |
| Chen et al | 2017 | * | * | * | * |  | * |  | * | * |  |  | 7 |  | Low |
| Das et al | 1986 | * | * |  | * |  |  |  | * | * |  |  | 5 |  | High |
| Delimaris et al | 2007 |  | * | * | * |  | * |  | * | * |  |  | 6 |  | Low |
| Gadomska et al | 1997 | * | * |  | * |  |  |  | * | * |  |  | 5 |  | High |
| Gadomska et al | 2005 | * | * |  | * |  | * |  | * | * |  |  | 6 |  | Low |
| Knapp et al | 2017 | * | * |  | * |  |  |  | * | * |  |  | 5 |  | High |
| Kuesel et al | 1992 |  |  |  | * |  | * |  | * | * |  |  | 4 |  | High |
| Melvin et al | 2012 | * | * | * | * |  | ** |  | * | * |  |  | 8 |  | Low |
| Qadir et al | 2007 |  | * |  |  |  |  |  | * | * |  |  | 3 |  | High |
| Yam et al | 1990 | * | * |  | * |  |  |  | * | * |  |  | 5 |  | Low |
| S1 - Is the Case Definition Adequate? S2 - Representativeness of the Cases, S3 - Selection of Controls, S4 - Definition of Controls,  C1 - Comparability of cases and controls on the basis of the design or analysis,  E1 - Ascertainment of exposure, E2 - Same method of ascertainment for cases and controls, E3 - Non-Response rate,  * indicates a point score  ROB – Risk of bias of each report; Low: ≥6 points , High: ≤5 points | | | | | | | | | | | | | | | |

|  |  |
| --- | --- |
| S1 | S2 |
|  |  |
| S3 | S4 |
|  |  |
| C1 | E1 |
|  |  |
| E2 | Overall Risk of Bias of all studies included |
| Supplementary Figure S2: Graphical illustration of the results of the critical assessment of studies; Is the Case Definition Adequate? (S1), representativeness of the Cases (S2), selection of Controls (S3), definition of Controls (S4), comparability of cases and controls on the basis of the design or analysis (C1), ascertainment of exposure (E1), same method of ascertainment of exposure for cases and controls (E2), overall risk of bias of all studies included. | |

| Supplementary Table S2: Sensitivity Analysis (using one study leave out method) of pooled mean differences of Lipid profiles between cases and non-cases of ovarian tumour | | | | | | | | | |
| --- | --- | --- | --- | --- | --- | --- | --- | --- | --- |
| **Study** | Year | **TC (***m*g/*d*L) | ***P*** | **TG (***m*mol/L) | ***P*** | **HDL (***m*mol/L) | ***P*** | **LDL (***m*mol/L) | ***P*** |
| None |  | -16.60 [-32.43, -0.77] | 0.04 | 0.06 [-0.19, 0.30] | 0.64 | -0.25 [-0.43, -0.08] | 0.005 | 0.11 [-0.24, 0.47] | 0.053 |
| Bukhari et al (A) | 2016 | -13.79 [-29.77, 2.18] | 0.09 | 0.02 [-0.16, 0.21] | 0.81 | -0.25 [-0.45, -0.06] | 0.01 | -0.01 [-0.35, 0.33] | 0.94 |
| Bukhari et al (B) | 2016 | -14.29 [-30.27, 1.69] | 0.08 | 0.06 [-0.20, 0.32] | 0.65 | -0.20 [-0.32, -0.07] | 0.002 | -0.06 [-0.35, 0.48] | 0.76 |
| Camuzcuoglu et al | 2008 | -16.12 [-32.40, 0.16] | 0.05 | 0.07 [-0.18, 0.32] | 0.59 | -0.26 [-0.45, -0.07] | 0.008 | 0.18 [-0.20, 0.55] | 0.36 |
| Chen et al | 2017 | § |  | 0.04 [-0.22, 0.29] | 0.77 | -0.25 [-0.48, -0.02] | 0.03 | § |  |
| Das et al (A) | 1986 | -15.78 [-31.98, 0.41] | 0.06 | § |  | § |  | § |  |
| Das et al (B) | 1986 | -15.69 [-31.87, 0.49] | 0.06 | § |  | § |  | § |  |
| Das et al (C) | 1986 | -16.22 [-32.38, -0.05] | 0.05 | § |  | § |  | § |  |
| Delimaris et al | 2007 | -18.37 [-34.51, -2.23] | 0.03 | § |  | -0.27 [-0.46, -0.09] | 0.004 | 0.06 [-0.31, 0.43] | 0.76 |
| Gadomska et al | 1997 | -14.85 [-31.09, 1.39] | 0.07 | 0.06 [-0.20, 0.31] | 0.66 | -0.23 [-0.41, -0.04] | 0.02 | § |  |
| Gadomska et al (A) | 2005 | -17.35 [-33.59, -1.11] | 0.04 | 0.06 [-0.19, 0.31] | 0.65 | -0.25 [-0.43, -0.06] | 0.01 | § |  |
| Gadomska et al (B) | 2005 | -16.39 [-32.71, -0.08] | 0.05 | 0.05 [-0.20, 0.31] | 0.68 | -0.23 [-0.41, -0.04] | 0.02 | § |  |
| Knapp et al | 2017 | -17.41 [-34.14, -0.69] | 0.04 | 0.06 [-0.21, 0.34] | 0.64 | § |  | § |  |
| Kuesel et al (A) | 1992 | -17.98 [-34.15, -1.81] | 0.03 | 0.04 [-0.21, 0.29] | 0.73 | § |  | § |  |
| Kuesel et al (B) | 1992 | -16.66 [-32.81, -0.51] | 0.04 | 0.03 [-0.21, 0.28] | 0.79 | § |  | § |  |
| Kuesel et al (C) | 1992 | -17.66 [-33.82, -1.50] | 0.03 | 0.04 [-0.21, 0.29] | 0.78 | § |  | § |  |
| Kuesel et al (E) | 1992 | -16.46 [-32.64, -0.28] | 0.05 | 0.07 [-0.18, 0.32] | 0.57 | § |  | § |  |
| Kuesel et al (F) | 1992 | -17.08 [-33.28, -0.88] | 0.04 | 0.07 [-0.18, 0.32] | 0.58 | § |  | § |  |
| Kuesel et al (G) | 1992 | -16.75 [-32.96, -0.54] | 0.04 | 0.08 [-0.17, 0.33] | 0.54 | § |  | § |  |
| Kuesel et al (I) | 1992 | -17.25 [-33.49, -1.00] | 0.04 | 0.06 [-0.19, 0.31] | 0.64 | § |  | § |  |
| Kuesel et al (J) | 1992 | -17.34 [-33.58, -1.09] | 0.04 | 0.05 [-0.20, 0.30] | 0.71 | § |  | § |  |
| Kuesel et al (K) | 1992 | -17.35 [-33.61, -1.09] | 0.04 | 0.06 [-0.19, 0.31] | 0.65 | § |  | § |  |
| Melvin et al | 2012 | -18.68 [-31.34, -6.02] | 0.004 | 0.06 [-0.22, 0.33] | 0.69 | -0.28 [-0.47, -0.09] | 0.004 | 0.06 [-0.44, 0.56] | 0.81 |
| Qadir et al | 2007 | -14.95 [-32.15, 2.24] | 0.09 | 0.10 [-0.07, 0.26] | 0.25 | -0.28 [-0.47, -0.09] | 0.004 | 0.17 [-0.21, 0.55] | 0.38 |
| Yam et al (A) | 1990 | -19.56 [-35.60, -3.52] | 0.02 | 0.06 [-0.19, 0.31] | 0.65 | -0.25 [-0.45, -0.06] | 0.009 | -0.01 [-0.38, 0.36] | 0.96 |
| Yam et al (B) | 1990 | -14.86 [-31.01, 1.29] | 0.07 | 0.08 [-0.17, 0.33] | 0.51 | -0.31 [-0.49, -0.13] | 0.0009 | 0.37 [0.03, 0.71] | 0.03 |
| §- the parameter was not assessed in the study.  *P-values* of studies that exerted negligible effect on the overall estimate of the mean estimate were marked in red. | | | | | | | | | |
