# Supplementary material for: Lipid profile and risk of ovarian tumours: a meta-analysis
Source: BMC Cancer. 2020 Mar 12;20:200. doi: 10.1186/s12885-020-6679-9 (PMC7068873; doi:10.1186/s12885-020-6679-9)
Supplement: Supplementary file 2 — Additional file 2. A Meta-analysis Of Observational Studies in Epidemiology (MOOSE) Checklist. [file 12885_2020_6679_MOESM2_ESM.doc]

| Meta-analysis Of Observational Studies in Epidemiology (MOOSE) Checklist | | | |
| --- | --- | --- | --- |
| **Criteria** | | | **A concise description of how the criteria were handled in the meta-analysis** |
| **Reporting of background should include** | | |  |
| √ | Problem definition | | Contemporary information from individual reports suggests inconsistent conclusion on the association between serum lipid profile and ovarian tumour (OT) risk. Our aim was to examine whether circulating lipid profile; total cholesterol (TC), triglyceride (TG), high-density lipoprotein (HDL) and low-density lipoprotein (LDL) differs between cases and non-cases of OT. |
| √ | Hypothesis statement | | Circulating lipid profile [total cholesterol (TC), triglyceride (TG), high-density lipoprotein (HDL) and low-density lipoprotein (LDL)] do not differ between cases and non-cases of OT |
| √ | Description of study outcomes | | Ovarian Tumour |
| √ | Type of exposure or intervention used | | Circulating lipid profile; total cholesterol (TC), triglyceride (TG), high-density lipoprotein (HDL) and low-density lipoprotein (LDL) |
| √ | Type of study designs used | | Observational studies |
| √ | Study population | | Human subjects |
| **Reporting of search strategy should include** | | |  |
| √ | Qualifications of searchers | | The qualifications of the investigators are specified in the author list. |
| √ | Search strategy, including time period included in the synthesis and keywords | | Medline, PubMed, EMBASE, Cochrane through January 2019.  Keywords: "lipid profile" OR "total cholesterol" OR "triglycerides" OR "high-density lipoprotein" OR "low-density lipoprotein" AND "ovarian cancer" OR "ovarian carcinoma" OR "epithelial ovarian cancer" OR " epithelial ovarian carcinoma" OR "ovarian benign tumour" OR "ovarian malignant tumour" OR "ovarian tumour". |
| √ | Databases and registries searched | | PUBMED, EMBASE, COCHRANE LIBRARY |
| √ | The search software used, name and version, including special features | | We did not use search software but employed EndNote to collate retrieved citations and remove duplications. |
| √ | Use of hand searching | | Bibliographies and supplementary materials of retrieved papers were searched by hand for additional references. |
| √ | List of citations located and those excluded, including justifications | | The detailed process of the literature search is outlined in the flow chart. Also, the list of citation is available on request. |
| √ | Method of addressing articles published in languages other than English | | Our search strategy was not limited with respect to language and time. Articles published in other languages were also considered. |
| √ | Method of handling abstracts and unpublished studies | | Abstracts were excluded. |
| √ | Description of any contact with authors | | None |
| **Reporting of methods should include** | | |  |
| √ | Description of relevance or appropriateness of studies assembled for assessing the hypothesis to be tested | | Detailed clarification of the inclusion and exclusion criteria was outlined in the method section. |
| √ | Rationale for the selection and coding of data | | Information such as; names of author, year of publication, country, study population, sample size of cases and control groups, lipid profile(s) determined, methods of analysis, criteria for defining cases and non-cases of ovarian tumours in each study; and the means values [alongside standard deviation (SD), standard error of mean (SEM)] of serum concentration of TC, and/or HDL and/or LDL and/or TG were independently extracted by two reviewers and inconsistencies in the data extractions were resolved in recourse to a third reviewer. To ensure uniformity of mean estimates, mean values of TC was transformed to (mg/*dL*), but TG, HDL and LDL were transformed to (mmol/*L*) and all values reported as SEM and CI were transformed to SD |
| √ | Assessment of confounding | | Sensitivity analyses were conducted by the one-leave-out method to evaluate the stability of the result. |
| √ | Assessment of study quality, including blinding of quality assessors; stratification or regression on possible predictors of study results | | Methodological assessment of included studies was conducted based on the Cochrane Handbook from the Cochrane Collaboration guidelines using the Newcastle-Ottawa Scale (NOS) for assessing the quality of observational studies in meta-analyses. Supplementary Material (SM) Tables S1, Figure S2 |
| √ | Assessment of heterogeneity | | Heterogeneity of the studies was determined using *I^2^*statistic. It provides the relative amount of variance of the summary effect as a result of the between-study heterogeneity. |
| √ | Description of statistical methods in sufficient detail to be replicated | | The method section contains a succinct explanation of the meta-analyses, sensitivity analyses and evaluation of the publication bias. |
| √ | Provision of appropriate tables and graphics | | 1 PRISMA Flow chart, 2 summary table, 4 forest plots of all studies, 4 funnel plots to examine publication bias, 1 table of sensitivity analyses. |
| **Reporting of results should include** | | |  |
| √ | | Graph summarizing individual study estimates and overall estimate | Figures 2, Tables S2 |
| √ | | Table giving descriptive information for each study included | Table 1 |
| √ | | Results of sensitivity testing | SM Tables S2 |
| √ | | Indication of statistical uncertainty of findings | Summary estimates and 95% confidence intervals, *P* values and results of sensitivity analyses were presented. |
| **Reporting of discussion should include** | | |  |
| √ | | Quantitative assessment of bias | Sensitivity analyses indicated there was no significant evidence of publication bias. |
| √ | | Justification for exclusion | Reports in pregnant and lactating women, as well as animals and cell studies, were excluded in the meta-analysis. Similarly, abstracts, reviews, letter to the editor and conference papers were also excluded. |
| √ | | Assessment of quality of included studies | Methodological assessment of included studies was conducted based on the Cochrane Handbook from the Cochrane Collaboration guidelines using the Newcastle-Ottawa Scale (NOS) for assessing the quality of observational studies in meta-analyses. Supplementary Material (SM) Tables S1, Figure S2 |
| **Reporting of conclusions should include** | | |  |
| √ | | Consideration of alternative explanations for observed results | We discussed that our meta-analyses had high I-squared statistics and studies were limited. |
| √ | | Generalization of the conclusions | We found TC and HDL profiles to be significantly lower in OT compared to non-OT subjects, and though TG and LDL profiles were different in OT compared to non-OT subjects, the difference was not statistically significant. |
| √ | | Guidelines for future research | The implications of HDL in tumour manifestations and growth need be validated in a large multi-ethnic longitudinal cohort and randomized clinical trials adjusting for relevant confounding factors. |
| √ | | Disclosure of funding source | Details of the funding sources for the study have been described in the funding section of the manuscript. |
